# Virucidal efficacy of a sonicated hydrogen peroxide system (trophon^®^ EPR) following European and German test methods

**DOI:** 10.3205/dgkh000287

**Published:** 2017-01-19

**Authors:** Britta Becker, Birte Bischoff, Florian H. H. Brill, Eike Steinmann, Jochen Steinmann

**Affiliations:** 1 Dr. Brill + Partner GmbH Institute for Hygiene and Microbiology, Bremen, Germany; 2 Institute for Experimental Virology, TWINCORE Centre for Experimental and Clinical Infection Research, Hannover, Germany

**Keywords:** disinfection, ultrasound probes, virucidal efficacy, trophon® EPR, human papillomaviruses

## Abstract

**Aim:** The virucidal efficacy of an automated ultrasound probe disinfector (trophon^®^ EPR) was evaluated in a three step procedure according to European and German test methods. This system uses sonicated hydrogen peroxide mist (35%) at elevated temperature (50°C) in a closed chamber with control of all parameters within a 7 minute cycle.

**Methods:** In the first step of examination, the peroxide solution was tested in a quantitative suspension assay according to the Guideline of Deutsche Vereinigung zur Bekämpfung der Viruskrankheiten (DVV) e.V. and Robert Koch-Institute (RKI) and in parallel with the European Norm EN 14476 with all test viruses creating a virucidal claim.

In the second step, the virucidal efficacy of the hydrogen peroxide solution was evaluated in a hard surface carrier test according to the Guideline of DVV with adenovirus, murine norovirus and parvovirus simulating practical conditions.

Finally, the efficacy was evaluated by the automated system using stainless steel carriers inoculated with test virus and positioned at different levels inside the chamber.

**Results:** A ≥4 log_10_ reduction of virus titre was demonstrated with all methods including carrier tests with murine norovirus, adenovirus, and parvovirus using the automated device.

**Conclusion:** The automated device is able to inactivate test viruses of German and European norms and can therefore claim efficacy against human pathogenic enveloped and non-enveloped viruses. This includes human papillomaviruses which form part of the complete virucidal claim.

## Zusammenfassung

**Zielsetzung:** Die viruziden Eigenschaften der maschinellen Aufbereitung von vaginalen Ultraschallsonden (trophon^®^ EPR) wurden in einem dreistufigen Prozess untersucht. Das System basiert auf der Vernebelung einer speziellen Peroxidlösung (35%) in einer geschlossenen Kammer bei erhöhter Temperatur (50°C) und einer Einwirkzeit von 7 min unter Kontrolle aller Parameter.

**Methode:** Im ersten Schritt wurde die Peroxidlösung in einem quantitativen Suspensionsversuch gemäß der Leitlinie der Deutschen Gesellschaft zur Bekämpfung von Viruskrankheiten e.V. (DVV) und des Robert Koch-Institutes und parallel gemäß der Europäischen Norm EN 14476 mit allen Prüfviren für eine viruzide Auslobung untersucht.

Im zweiten Schritt folgte ein Keimträgerversuch entsprechend der DVV-Leitlinie mit murinem Noro-, Adeno- und Parvovirus, um eine Viruzidie „high level“ zu zeigen.

Abschließend wurde die Wirksamkeit der maschinellen Aufbereitung mit den Viren der DVV-Leitlinie auf viruskontaminierten Edelstahlträgern überprüft.

**Ergebnisse:** Eine ≥4 log_10_ Reduktion des Virustiters konnte mit allen Methoden einschließlich der maschinellen Aufbereitung mit murinem Noro-, Adeno- und Parvovirus erreicht werden.

**Schlussfolgerung:** Die maschinelle Aufbereitung ist in der Lage, die Prüfviren der Deutschen und Europäischen Normen zu inaktivieren und erzielt damit den Anspruch, alle humanpathogenen behüllten und unbehüllten Viren einschließlich der humanen Papillomaviren zu inaktivieren, die Teil dieses resultierenden Claims einer kompletten Viruzidie sind.

## Introduction

Ultrasound probes in contact with mucous membranes and blood are classified as semi-critical devices and must be sufficiently disinfected to inactivate bacteria, fungi, and viruses. A review has shown that despite the use of a protective sheath, intracavity probes can be contaminated with human immunodeficiency virus (HIV), cytomegalovirus (CMV), herpes simplex virus (HSV) and human papillomavirus (HPV) [1]. Among HPV certain high risk types like HVP16 and HPV18 are associated with cervical neoplasia. Several studies have shown intracavity ultrasound probes are contaminated with HPV following low level disinfection, and the virus can also be found throughout the gynaecological environment [2], [3], [4], [5], [6]. In a study with native HPV16 virions, it was shown that glutaral (GTA) and *ortho*-phthalaldehyde (OPA) were unable to inactivate the virus while hypochlorite and a peracetic acid (PAA)-silver based disinfectant were effective [7].

Nowadays, many countries mandate high level disinfection of semi-critical ultrasound probes naming disinfectants such as GTA, OPA, and hydrogen peroxide with virucidal efficacy [8], [9], [10]. Both the probe body and handle should undergo high-level disinfection [11]. 

Disinfection methods presumably vary in many countries ranging from manual disinfection methods with impregnated wipes using quaternary ammonium compounds (QAC) to automated methods [12]. Recently, an automated sonicated hydrogen peroxide device (trophon^®^ EPR) has demonstrated its efficacy against native HPV16 and HPV18 virions [13]. 

In this study we examined the virucidal efficacy of the hydrogen peroxide solution used in the automated device with viruses of German and European Guidelines in phase 2/step 1 (suspension assay) and phase 2/step 2 (carrier assay) testing. Finally, contaminated stainless steel carriers in clean conditions were evaluated in the automated device under normal use conditions according to manufacturer instructions. Complete inactivation of these mandated test viruses would permit a virucidal claim against all enveloped and non-enveloped viruses including HPV in Germany and Europe [14]. 

## Materials and methods

### Study design

The whole study was based upon a three step procedure. In the first two steps the hydrogen peroxide solution used in the automated device was evaluated in a quantitative suspension test (phase 2/step 1) and a carrier test simulating practical conditions (phase 2/step 2) as required by German Guidelines and the European Norm. The third step involved placing carriers inoculated with test virus inside the automated device chamber. The automated device is designed for the disinfection of intracavity ultrasound probes using sonicated, nebulised hydrogen peroxide in a 7 min cycle.

### Viruses

The poliovirus type 1 strain LSc-2ab (Chiron-Behring) was obtained from PD Dr. O. Thraenhart, Eurovir, D-14943 Luckenwalde. The adenovirus (AdV) type 5 strain Adenoid 75 was obtained from PD Dr. A. Heim, Institute of Medical Virology, Hannover Medical School, D-30625 Hannover. The vaccinia virus strain Elstree (VR-1549, ATCC) originated from the Institute of Medical Virology and Immunology of the University of Essen, D-45122 Essen. Polyomavirus SV40 strain 777 originated from PD Dr. A. Sauerbrei, Institute of Virology and Antiviral Chemotherapy at the Friedrich Schiller University of Jena. The murine parvovirus (Minute Virus of Mice, MVM) was obtained from the Paul-Ehrlich-Institute, D-63225 Langen. Murine norovirus S99 (MNV) was obtained from PD Dr. E. Schreier at the Robert Koch-Institute (RKI) in D-13302 Berlin. 

### Virus propagation and cell culture

The test virus suspensions were prepared by infecting monolayers of the respective cell lines. The virus titres of these suspensions ranged from 10^7^ to 10^9^ TCID_50_/mL. Poliovirus type 1 was propagated in BGM cells (buffalo green monkey kidney cell line; supplied by Prof. Dr. Lindl, Institute for Applied Cell Culture, D-81669 München) and AdV type 5 in A549 cells (human lung epithelial carcinoma cells). The A549 cells originated from the Institute of Medical Virology, Hannover Medical School, D-30625 Hannover. Vaccinia virus strain Elstree replication was performed in Vero cells (monkey kidney cell line) obtained from Vircell, SL in ES-18329 Santa Fe, Spain (now BIOTRIN International GmbH, D-69126 Heidelberg). Polyomavirus SV40 strain 777 was propagated in CV-1 cells (kidney cells of African green monkey) and MVM strain Haden in A9 cells (mouse cell line, originated from Paul-Ehrlich-Institute, D-63225 Langen). MNV strain S99 was propagated in RAW 264.7 cells (a macrophage-like, Abelson leukemia virus transformed cell line derived from BALB/c mice, ATCC TIB-71). Poliovirus and MNV were replicated in Dulbecco’s Modified Eagle’s Medium (DMEM), all other viruses in Eagle’s Minimum Essential Medium with Earle’s BSS (EMEM).

### Sonicated hydrogen peroxide system

The sonicated hydrogen peroxide system (trophon^®^ EPR) is an automated high level disinfection device designed for ultrasound probes. The hydrogen peroxide solution is supplied in an enclosed cartridge (NanoNebulant; 34.9–37.0% hydrogen peroxide, batch number A202011) which is pierced open once positioned and closed inside the custom built compartment. The device used had serial number 29709-026 and was manufactured by Nanosonics Limited (Lane Cove West, New South Wales, Australia).

### Quantitative suspension test (Guideline of DVV/RKI)

Tests were carried out according to the DVV/RKI Guideline at 20°C with poliovirus, adenovirus (AdV), polyomavirus SV40 and vaccinia virus [15]. One part by volume of test virus suspension and one part by volume of double distilled water or FCS were mixed with eight parts by volume of the formulation. Due to high cytotoxicity NanoNebulant was diluted to 60%, 40% and 10% (demonstration of the non-active range) with double distilled water. Infectivity was stopped by immediate serial dilution with ice-cold medium and later determined by means of end point dilution titration in microtitre plates. 100 µl of each dilution were placed in eight wells of a sterile polystyrene flat bottomed 96-well microtiter plate containing 100 µl cell suspension. Cultures were observed for cytopathic effects (CPE) after 4–18 days of inoculation depending on the cell culture system. Tests according to the German Guideline were conducted in two independent test runs on different days. Virus controls were incorporated after the longest exposure time. Besides the end point dilution method the large-volume-plating (LVP) method as described in the German Guideline was used [16]. Furthermore, MicroSpin columns (MicroSpinTM S-400 HR columns, GE Healthcare, Freiburg, Germany) were introduced as described in the EN 14476 when testing vaccinia virus strain Elstree and polyomavirus SV40 strain 777 [17]. For determination of cytotoxicity the formulations were serially diluted 10-fold in DMEM up to a dilution of 10^–5^. One part by volume of water of standardized hardness (instead of test virus suspension) was mixed with one part by volume of interfering substance and eight parts by volume of the disinfectant. Aliquots of 100 µl of each test concentration and each dilution were then inoculated into eight wells of a 96-well microtiter plate containing 100 µl cell suspension. The cell cultures were observed for cytotoxic effects for the same incubation time as afterwards used for the quantitative suspension tests. Virus titres were determined using the methods of Spearman [18] and Kaerber [19] and expressed as log_10_ TCID_50_/ml including standard deviation. Titre reduction is presented as the difference between the virus titre after the exposure time with the disinfectant and the control virus titre (water). According to the Guideline of the DVV/RKI, a formulation under test conditions must give at least a 4 log_10_ reduction in infectivity titre of test virus (inactivation = 99.99%) at the recommended concentration and exposure time to be considered active [15]. Different controls like cytotoxicity, interference and sufficient suppression of virucidal activity were additionally included. 

### Quantitative suspension test according to EN 14476

Tests according to EN 14476 were run in parallel to the Guideline of DVV/RKI with poliovirus, AdV and murine norovirus (MNV) as test viruses of the EN 14476 and the corresponding permissive cells [17]. The main difference to the German DVV/RKI Guideline is the change from double distilled water and FCS as interfering substances to clean conditions (0.3% bovine serum albumin, final concentration in the test procedure 0.3 g/l). Again, all controls as described in EN 14476 were included [17]. 

### Quantitative carrier test

The quantitative carrier test according to the Guideline of DVV was performed in clean conditions with AdV, MNV and Minute Virus of Mice (MVM) [20]. The peroxide solution was again diluted with water due to high cytotoxicity. The cleaning of the stainless steel discs (20 mm diameter, GK Formblech GmbH, D-12277 Berlin, Germany) was performed as described in the Guideline [20]. A total of 50 µl of the virus inoculum was deposited on each pre-treated carrier and dried. Then, inoculum was covered with 100 µl of the peroxide solution. Immediately at the end of the exposure time, the discs were transferred into plastic vial holders (Sarstedt AG & Co. KG, D-51582 Nümbrecht) with 9,900 ml of ice-cold culture medium to stop the activity of the formulation. Vials were vortexed for 1 min to recover the residual viruses and the eluate was immediately diluted 10-fold (quantal test method) for determining viral infectivity. For MVM the LVP method instead of end point dilution was used. Cytotoxicity was measured as described in the DVV Guideline [20]. 

### Test in the automated device

**Figure 1:**
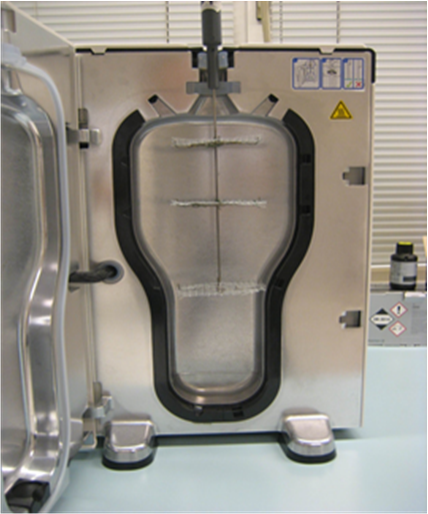
Automated testing device with stainless steel discs contaminated with test virus suspension. Three carriers with virus were run in parallel. A fourth carrier without virus is measuring the cytotoxicity in the test system.

For each test virus three stainless discs as described in the carrier test according to the Guideline of DVV were prepared plus control discs and placed at different levels in the device (top – middle – bottom). The inactivation experiments were run in two independent assays on two different days. Test discs were placed aseptically in a Petri dish and inoculated with 50 µl of the virus inoculum [20]. After drying, the discs were placed in an attachment simulating the probe (Figure 1 (Fig. 1)). In different runs, three discs were placed in the top, the middle and the bottom of the attachment. After each run, the action of the product on the carrier was stopped by immediate dilution with 10 ml ice-cold medium in a separate container (25 ml with cap). The container was vortexed for 60 seconds to re-suspend the virus. Directly after elution, series of ten-fold dilutions of the eluate in ice-cold maintenance medium were prepared and inoculated on cell culture. Titration of the virus controls was performed before drying and after drying (threefold assay) outside of the device at room temperature. Determination of cytotoxicity was performed as described in the Guideline of DVV [20]. Stainless steel discs were inoculated with 50 µl cell culture medium (without FCS) instead of the virus inoculum. Furthermore, a cell control (only addition of medium) was incorporated [20]. 

## Results

### Quantitative suspension test (phase 2/step 1)

The hydrogen peroxide solution was examined in the suspension test according to German and European Standards. Due to high cytotoxicity the ready-to-use product was diluted with distilled water to be able to demonstrate a 4 log_10_ reduction. For the German Guideline of DVV/RKI the requested decline of 4 log_10_ steps was demonstrated for poliovirus, AdV and SV40 with the 60% dilution after 30 seconds exposure time and for vaccinia virus with the 40% solution (Table 1 (Tab. 1)). The mean reduction factors (RF) were ≥5.50 ± 0.19 (Aqua bidest.), ≥5.25 ± 0.22 (FCS) for poliovirus and ≥4.82 ± 0.24 (Aqua bidest.), ≥4.50 ± 0.16 (FCS) for AdV and ≥4.25 ± 0.33 (Aqua bidest.) and ≥4.32 ± 0.34 (FCS) for SV40. Testing vaccinia virus the 40% solution was active after 30 seconds exposure time with RF of ≥4.25 ± 0.23 (Aqua bidest.) and ≥4.25 ± 0.23 (FCS). In addition, the peroxide was tested against poliovirus, AdV and MNV according the EN 14476 in clean condition. The following RF resulted after one minute exposure time: poliovirus ≥4.88 ± 0.18, AdV ≥5.00 ± 0.33 and MNV ≥4.00 ± 0.25 (Table 2 (Tab. 2)). In all cases no residual test virus could be detected. 

### Quantitative carrier test (phase 2/step 2)

In the next step, the peroxide solution was examined in a phase 2/step 2 according the Guideline of DVV with two independent runs with three carriers (Table 3 (Tab. 3)). AdV, MNV and MVM served as test viruses. Since the transvaginal ultrasound probes in the automated device are treated at 50°C, these tests were performed at both 20°C and 50°C. Against AdV the formulation was active as a 60% solution after three minutes at 20°C (RF ≥4.02 ± 0.36) and at 50°C (≥4.13 ± 0.43). For MNV the 60% solution was active reaching RF of ≥4.09 ± 0.26 at 20°C, and the 10% solution reached a RF of ≥4.88 ± 0.22 at 50°C. For MVM undiluted application was necessary at 20°C and 50°C with three minutes exposure time using the LVP method (RFs 4.03 and 4.05). 

### Quantitative field test (metal carriers inside device)

Finally, identical carriers were run in the automated device simulating practical conditions with AdV, MNV and MVM in clean conditions. In all cases, a RF greater than 4 (inactivation 99.99%) could be detected (Table 4 (Tab. 4)).

## Discussion

Different studies have shown that intracavity ultrasound probes are contaminated after low-level disinfection with pathogenic bacteria, fungi and viruses including HPV [1], [3], [4]. One study found 7.5% and 18.4% of the surveillance samples being contaminated with HPV after using a quaternary ammonium compound based agent [2]. In another study looking at ultrasound probes in clinical use, HPV could not be detected on 50 probes after disinfection with a chlorine dioxide based wipe [21]. 

In the present study, we have used suspension, carrier and simulated use tests to show a greater than 4 log_10_ reduction in viral infectivity. Such tests with high viral titre are required to claim virucidal efficacy. Therefore, official authorities in many countries are requesting a high-level disinfection for transvaginal probes [8], [9], [10]. Data are available that automated devices can reduce contamination of intracavity ultrasound probes to background levels [5]. One study showed that the automated device could reduce contamination with a success rate of 91.4% while a manual wipe method showed greater contamination (success rate of 78.8%) based on a study measuring bacterial contamination rates [12]. When asking sonographers about high-level disinfection with the automated device and glutaral-based soak systems, a higher level of satisfaction was reported for the automated system [22]. This fully-automated high-level disinfection system for ultrasound probes is therefore strongly recommended by several authors [23], [24]. 

In our study well established Guidelines from Europe were incorporated in a stepwise approach with chosen test viruses being able to generate a general virucidal claim. This virucidal claim extends to all enveloped and non-enveloped viruses since the tests are based on different viruses from representative virus families. In the suspension test very stable test viruses were included like poliovirus, AdV and SV40, which is regarded as a surrogate virus for HPV because historically they were grouped together in the same no longer existing virus family papovaviridae [14]. In the carrier test AdV, MNV and MVM were chosen from the German Guideline of DVV [20] showing that MVM proved to be the most stable virus here. 

From the clinical point of view the focus must be on HPV. Unfortunately, HPV cannot be cultivated with traditional cell culture techniques. But it is well known that HPV is associated with human cancer. An organotypic raft culture technique has been developed allowing HPV replication in terminally differentiated keratinocytes [7]. Data from this model has demonstrated that GTA and OPA were not able to inactivate HPV16 in a suspension assay, whereas hypochlorite and PAA-silver succeeded [7]. Later the same research group demonstrated complete HPV inactivation using the automated device in a hard surface carrier test [13]. 

In our study when using representative test viruses from different virus families and a stepwise approach including suspension and hard surface carrier tests, as well as a test in the automated device, the same conclusion can be drawn as with the examination done with HPV as sole test virus. A virucidal claim (including HPV efficacy) results from suspension and carrier tests including testing in the automated device. 

## Notes

### Competing interests and funding

Nanosonics Limited funded the study and provided the device for testing virus inactivation.

E. S. was supported by the Helmholtz Centre for Infection Research. 

## Figures and Tables

**Table 1:**
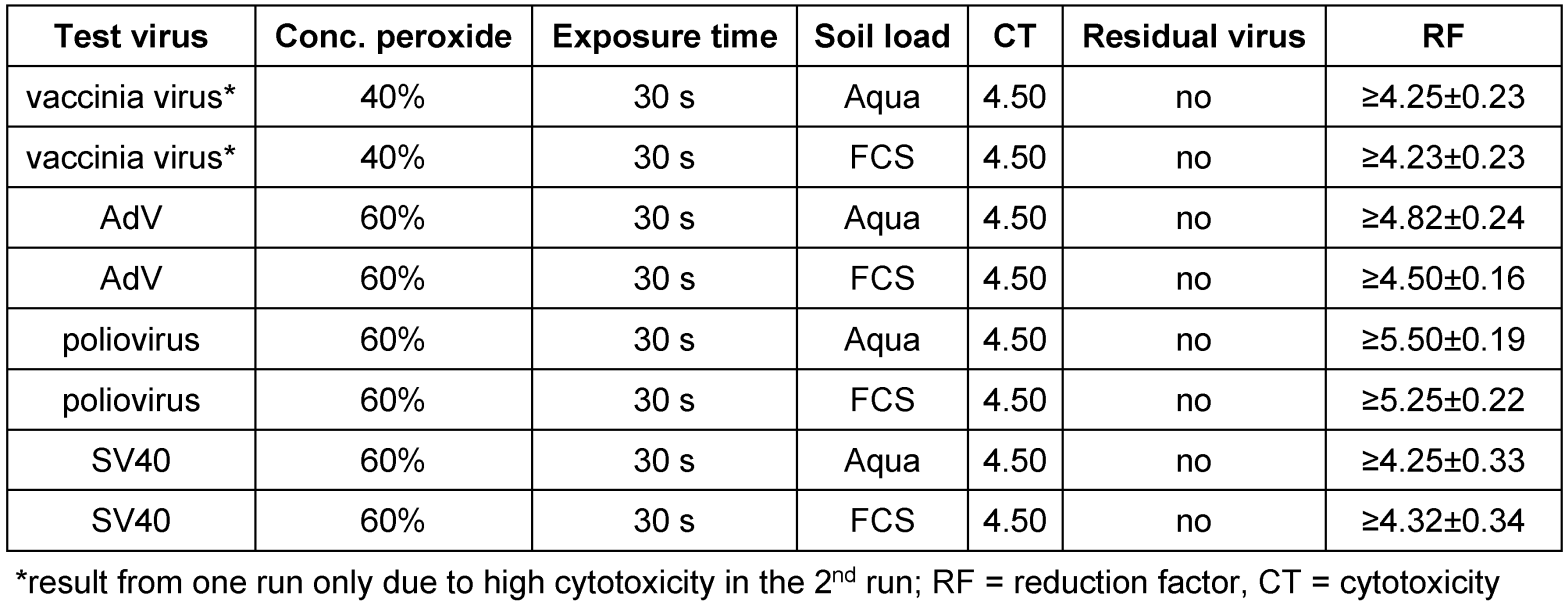
Activity of hydrogen peroxide solution in the quantitative suspension test under clean conditions (20°C) against vaccinia virus, adenovirus, poliovirus and polyomavirus SV40 in accordance with the Guideline of DVV/RKI. Results are derived from the quantitative suspension test in duplicates and presented as reduction factor (RF) with 95% confidence interval (data with SV40 and vaccinia virus with columns).

**Table 2:**
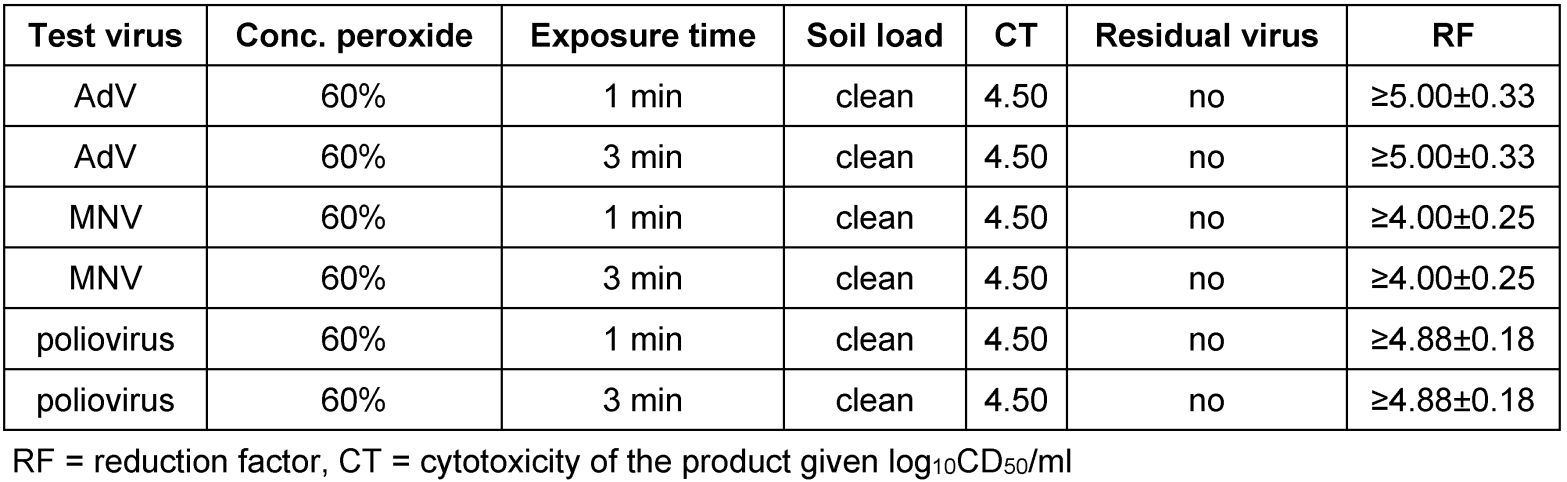
Activity of hydrogen peroxide solution in the quantitative suspension test under clean conditions against adenovirus, murine norovirus and poliovirus according to EN 14476. The concentration of the product was reduced to 60% due to the high cytotoxicity. Results presented as reduction factor (RF) with 95% confidence interval.

**Table 3:**
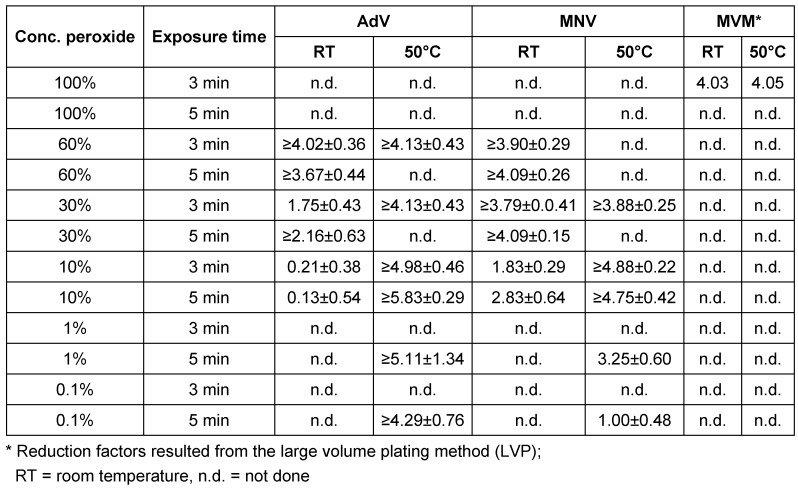
Efficacy of hydrogen peroxide solution in the carrier test under clean conditions against adenovirus, murine norovirus and murine parvovirus (Minute Virus of Mice = MVM) according to the Guideline of DVV. Results are presented as reduction factor (RF) with 95% confidence interval.

**Table 4:**
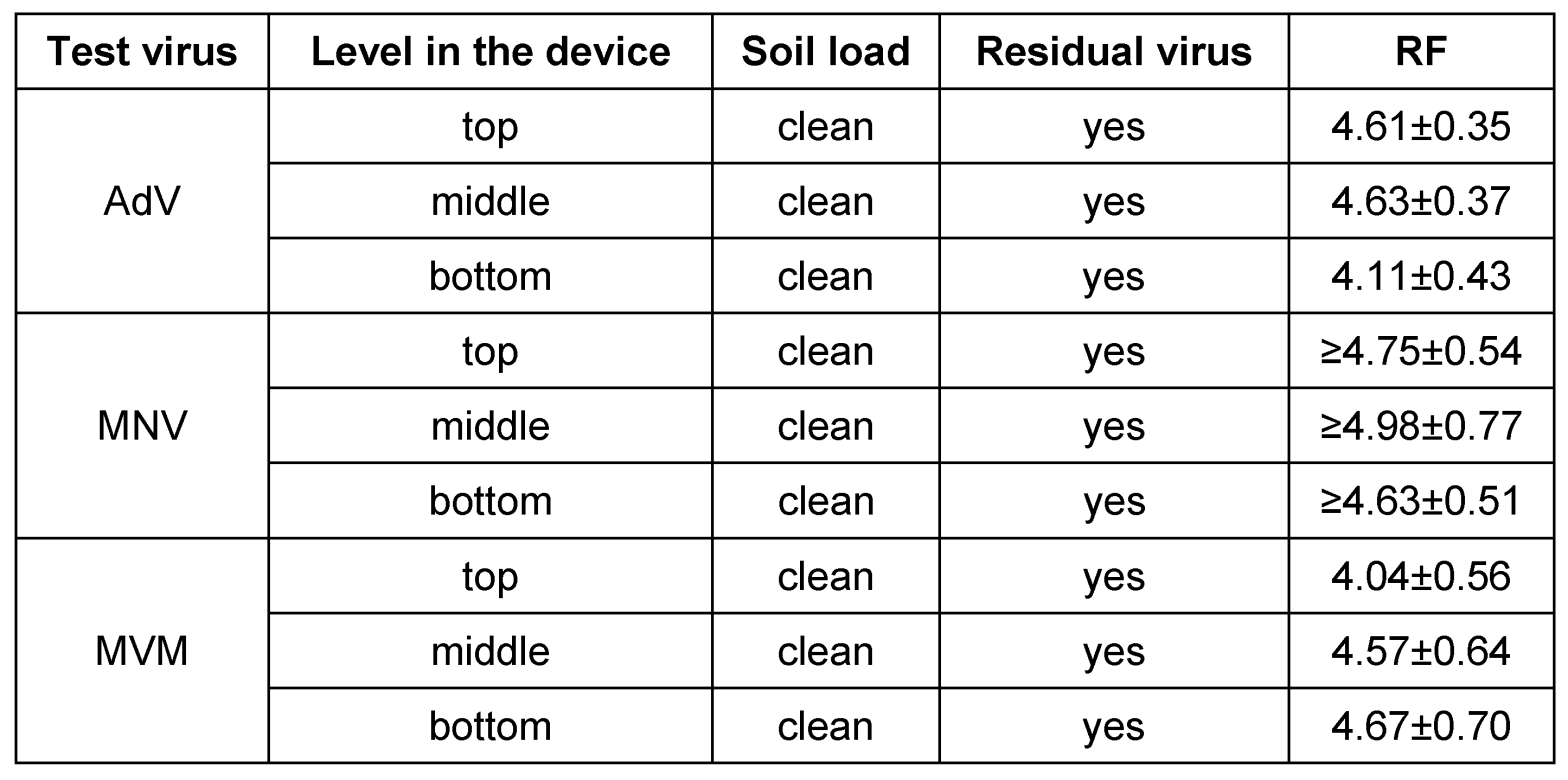
Efficacy of the automated device with stainless steel carriers contaminated with virus inoculum against adenovirus, murine norovirus and murine parvovirus (Minute Virus of Mice = MVM). The run took seven minutes at a temperature of 50°C. Data are given as reduction factor (RF) with 95% confidence interval.
